# The Avocado Sunblotch Viroid: An Invisible Foe of Avocado

**DOI:** 10.3390/v11060491

**Published:** 2019-05-29

**Authors:** José Ramón Saucedo Carabez, Daniel Téliz Ortiz, Moisés Roberto Vallejo Pérez, Hugo Beltrán Peña

**Affiliations:** 1 Departamento de Investigación Aplicada-Driscolls, Libramiento Sur #1620, Jacona 59833, Michoacán, Mexico; 2 Postgrado en Fitosanidad-Fitopatología, Colegio de Postgraduados, Km. 36.5, Montecillo, Texcoco 56230, Estado de México, Mexico; dteliz@hotmail.com; 3 Consejo Nacional de Ciencia y Tecnología-Universidad Autónoma de San Luis Potosí, Álvaro Obregón #64, San Luis Potosí 78000, San Luis Potosí, Mexico; vallejo.pmr@gmail.com; 4 Departamento de Ciencias Naturales y Exactas-Fitopatología, Universidad Autónoma de Occidente UR Los Mochis, Boulevard Macario Gaxiola, Los Mochis 81223, Sinaloa, Mexico; hugocheves@hotmail.com

**Keywords:** avocado, *Persea americana*, avocado sunblotch viroid

## Abstract

This review collects information about the history of avocado and the economically important disease, avocado sunblotch, caused by the avocado sunblotch viroid (ASBVd). Sunblotch symptoms are variable, but the most common in fruits are irregular sunken areas of white, yellow, or reddish color. On severely affected fruits, the sunken areas may become necrotic. ASBVd (type species *Avocado sunblotch viroid*, family *Avsunviroidae*) replicates and accumulates in the chloroplast, and it is the smallest plant pathogen. This pathogen is a circular single-stranded RNA of 246–251 nucleotides. ASBVd has a restricted host range and only few plant species of the family *Lauraceae* have been confirmed experimentally as additional hosts. The most reliable method to detect ASBVd in the field is to identify symptomatic fruits, complemented in the laboratory with reliable and sensitive molecular techniques to identify infected but asymptomatic trees. This pathogen is widely distributed in most avocado-producing areas and causes significant reductions in yield and fruit quality. Infected asymptomatic trees play an important role in the epidemiology of this disease, and avocado nurseries need to be certified to ensure they provide pathogen-free avocado material. Although there is no cure for infected trees, sanitation practices may have a significant impact on avoiding the spread of this pathogen.

## 1. The Origin of the Avocado and the Avocado Sunblotch Viroid

**Figure 1:**
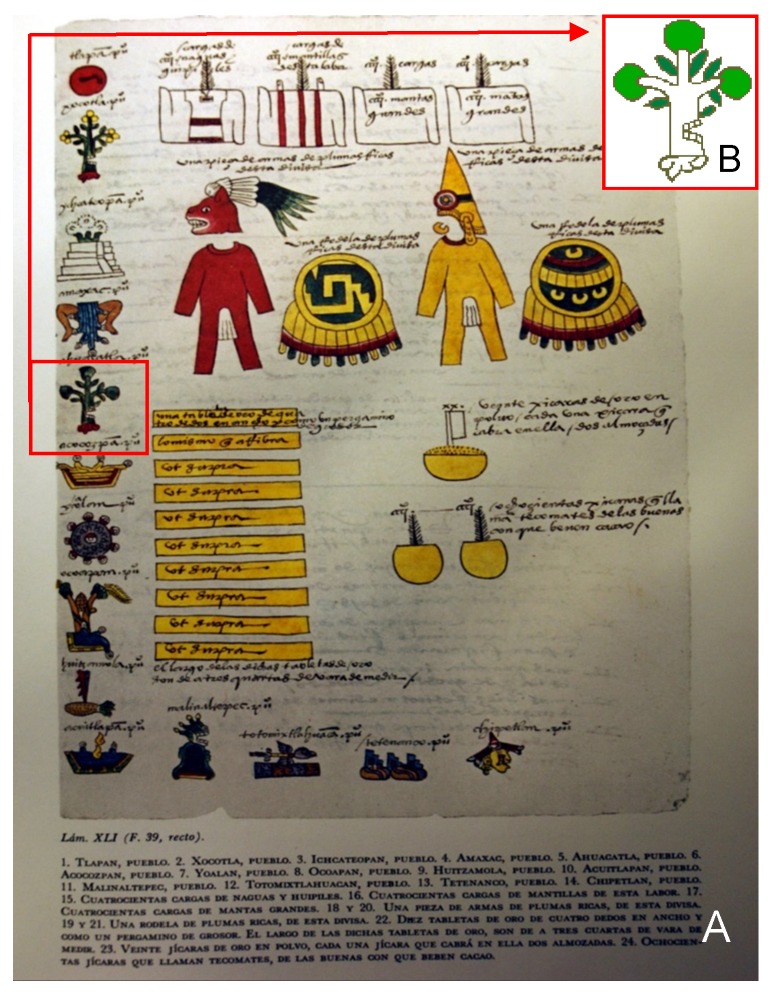
Pictographic script from the Mendoza Codex (**A**) with an illustration of Ahuacatlan town represented by an avocado tree (**B**) (fifth from top left). The teeth drawing on the trunk means “place”. Ahuacatlan means “place where avocados grow”.

Mesoamerica is considered the center of origin of the avocado, principally the highlands of México and Guatemala [1]. The oldest avocado fossils, dating back more than 8000 years ago [2], were found in the Caves of Coxcatlán, in the Tehuacán Valley of the State of Puebla, México. Human pilgrimages extended it to the South of México and North of Central America, down to Colombia, Venezuela, Ecuador, and Perú [3]. Ahuacatl (aguacate) in the Nahuatl language means “testicle”, from the shape of the fruit. Domesticated avocado seeds were found buried with Incan mummies in Perú dating back to 750 B.C., and there is evidence that avocados were cultivated in México as early as 500 B.C. [4]. The Mendoza Codex was painted by Mexican painters with the intent by México’s first virrey, Antonio de Mendoza, to send it to Carlos I, King of Spain (also Charles V of the Holy Roman Empire). This codex is a pictographic script, which in the first chapter describes life in México from 1325 (the foundation of Tenochtitlan, the great Mexica Empire Capital) to the arrival of the Spanish invasion (1521). The second chapter lists all the towns in Mexica´s domain and the tributes they should periodically deliver. The folio (page 39) lists 14 towns, the fifth of which (from the top left) is Ahuacatlan, represented by an avocado tree (Figure 1). The teeth drawing on the trunk means “place”, and Ahuacatlan means “place where aguacate (avocado) grows”. There are several Ahuacatlan towns in the States of México, Nayarit (Ahuacatlan), and Puebla, and in Guatemala and Colombia. The 14th month in the Mayan calendar (K’ank’in) is represented by an avocado glyph [3]. During the reign of Carlos I, the Crown of Spain (Castilla) expanded its territories over much of America, and avocado was probably spread during this expansion: Hernán Cortés conquered the Mexica Empire in 1521, which would result in the Kingdom of New Spain (Spaniards tried avocados for the first time), and Nuño de Guzmán conquered the Tarascan Empire and founded the Kingdom of Nueva Galicia. The first written document that mentions aguacate is by Francisco Fernández de Enciso, who found and tasted aguacate in Yaharo, close to Santa Marta, Colombia. He described the fruit: “it looks like an orange and the pulp is yellow, like butter with a delicious, soft and marvelous taste” [3]. Palta is another name in Spanish used in South America for aguacate. Pedro de Cieza described in 1532–1550 the presence of avocado in several countries. He mentioned aguacate as the fruit that belongs to Panama. Then, he mentioned it as palta in Colombia (Cártago, Cali, and Cauca Valley) and Ecuador. Garcilaso de la Vega in “Comentarios regios de los Incas” mentioned Tupac Inco Yupanqui advancing and conquering several towns (1450–1475), one of which was Palta, mentioning that exquisite fruit named palta. This is the approximate time when the palta tree was brought from Ecuador to Perú. In Europe, the avocado was first mentioned by Clusius, who referred to avocado trees of a Mexican type growing in Valencia, Spain. Now, avocados are cultivated and consumed on the five continents of our planet [3].

The sunblotch disease is economically important in avocado (*Persea americana* Miller) [3,5,6] and is a quarantine pest in some countries [7]. The sunblotch was observed first in Southern California in 1914 and Palestine in 1924 [8]. The first official report of sunblotch was published in 1928 in the USA, and the symptoms were attributed to physiological causes (solar irradiation) [9] or to a genetic disorder [7], and to a virus following graft-transmissibility [8,10,11,12]. Since virus particles were not observed by electron microscopy in sections or extracts from tissue affected by sunblotch and since low-molecular-weight RNA was isolated from these tissues, a viroid was suggested as the potential causal agent of this disease [13,14,15]. A viroid was indeed purified from avocado leaves infected by sunblotch disease and named avocado sunblotch viroid [16,17]. ASBVd was found to be associated with avocado sunblotch disease and was characterized as a covalently closed circular RNA molecule with a molecular size lower than the chrysanthemum stunt viroid and citrus exocortis viroid, while hybridization analysis with 32P-labeled complementary DNA indicated that the viroid consists of a single RNA species [18]. ASBVd was confirmed in 1981 as the causal agent of the disease when healthy avocado seedlings developed symptoms of avocado sunblotch disease 2–5 months after being inoculated with bark from infected trees or 4–8 months later when inoculated with filter paper pieces moistened with the purified viroid. Infection by ASBVd was confirmed in seedlings inoculated by both methods by PAGE and cDNA probe [16]. In 1948, in a tour to México, in the Rodiles grove near Atlixco, State of Puebla, a commission of The California Avocado Society noted avocado trees with sunblotch [19]. Actually, the ASBVd origin has not been confirmed, but it is known that the avocado industry in Southern California began with trees and seeds from México and Guatemala, and that the cities of Atlixco and Queretaro, México, were two important sources of seeds [8]. This germplasm could have been infected with ASBVd. This evidence suggests that the ASBVd origin is the same as that of its natural host [7,8]. Considering the molecular nature of ASBVd and its dependence on its natural host, the avocado, we postulate that the viroid evolved together with the avocado plant a long time ago.

## 2. Symptoms

**Figure 2:**
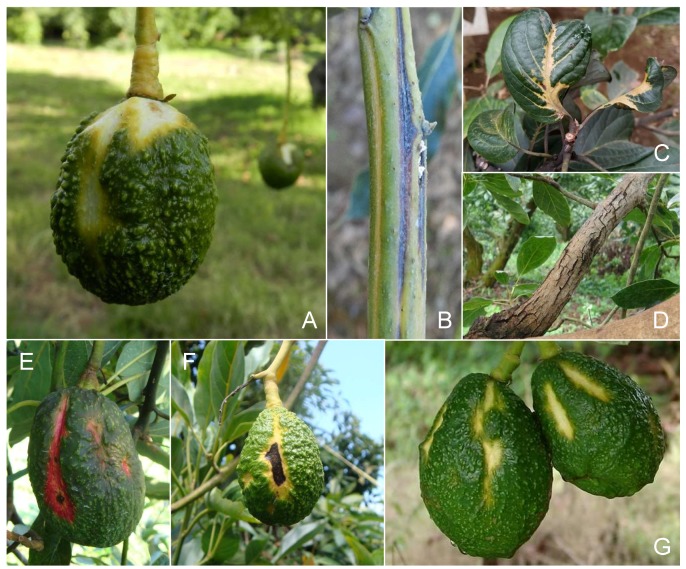
Symptoms of avocado sunblotch disease. Yellowish sunken areas on fruits (**A**); discolored and necrotic depressions on infected twigs (**B**); distortion and variegation on leaves (**C**); cracked bark (“Alligator skin”) appearance on some mature branches (**D**); fruits with reddish color areas (**E**); necrosis on severely affected fruits (**F**); multiple yellowish sunken areas in fruits (**G**).

The alterations caused by ASBVd vary and are influenced by the cultivar, environmental conditions, and the variants of the viroid that predominate in the host [20,21,22,23,24]. The most typical symptom on fruits is sunken crevices of white, yellow, or reddish color (Figure 2A) [25,26]. On severely affected fruits, the sunken areas may become necrotic (Figure 2F) [27,28]. The bases of some shoots and young branches of infected trees may show discolored streaks or stripes (Figure 2B). On some leaves of infected trees, distorted and variegated areas develop from the central vein that may progress and deform the entire leaf blade (Figure 2C). The bark of some trunks and old branches of some infected trees develop a cracked appearance also known as “alligator skin” (Figure 2D). The distribution of sunblotch symptoms is irregular, and infected trees may develop only one or multiple symptoms. In some cases, infected trees are fruitless and remain stunted [26,29,30]. The most reliable ASBVd symptom in México is the sunken crevices of white, yellow, or reddish color on fruits (Figure 2E). In some cases, multiple fruits with initial symptoms may occur on the same tree (Figure 2G). Remarkably, some infected trees are asymptomatic but may develop symptoms under stress conditions. Likewise, symptomatic trees may become asymptomatic for underexplored reasons. More research is needed to understand the distribution and possible variants associated with the different symptoms.

## 3. Taxonomy and Structure of ASBVd

As stated by Flores et al., viroids are subviral plant pathogens at the frontier of life [31]. They are solely composed by a single-stranded circular RNA of 246–434 nt with a compact secondary structure. Some properties of viroids, particularly the presence of ribozymes in members of the family *Avsunviroidae*, suggest that they might have appeared very early in evolution and could represent “living fossils” of the precellular RNA world that presumably preceded our current world based on DNA and proteins [31,32,33]. Viroids replicate autonomously when inoculated into their host plants and incite, in most of them, economically important diseases. The characterized viroids are exclusive to the plant kingdom, and analysis of their structural and functional properties has grouped them into two families: *Pospiviroidae* (type species *Potato spindle tuber viroid*) and *Avsunviroidae* (type species *Avocado sunblotch viroid*) (ASBVd) [31]. ASBVd is a plant pathogen that affects avocado and other members of the *Lauraceae* [23]. ASBVd replicates and accumulates in the chloroplasts, and the single-unit nuclear-encoded polymerase (NEP) is the RNA polymerase required in ASBVd replication [34]. ASBVd also presents the smallest genomic size (246–250 nt) and an A + U content (62%) above that of any other viroid (40–47%) [35], with this characteristic suggesting a polyphyletic viroid origin [31]. Recently, Giguère et al. [36] studied the structure features of the ASBVd with a high-throughput technique of selective 2′-hydroxyl acylation analyzed by primer extension (hSHAPE) [36]. Although they did not investigate the structure of multiple variants of the ASBVd, with this approach they provided some features not elucidated before: A loop in the left and the terminal domain as well as a central loop with the conserved hammerhead nucleotides in the rod-like structure [36]. The mechanism of ASBVd replication that operates is the symmetric rolling circle, where the monomeric circular form (mc) (+) of RNA serves as a template for the synthesis of the oligomeric head-to-tail (-) RNA intermediates that self-cleavage into monomeric linear forms (ml) (-) and are subsequently circularized to act as the initial template for the second half of the cycle [37] involving the single-unit nuclear-encoded polymerase [34]. The self-cleavage is mediated by cis-acting hammerhead ribozymes embedded in both polarity RNAs [38,39].

## 4. Host range of ASBVd

ASBVd belongs to the *Avsunviroidae* family and has one of the narrowest host ranges among the viroids [23,24]. Experimentally, ASBVd was transmitted first to *Cinnamomum zeylanicum* plants that expressed typical symptoms of yellow depressed streaks on the stem [40,41] and subsequently to *Persea schiedeana*, *Ocotea bullata*, and *Cinnamomum camphora* [42], all belonging to the family *Lauraceae*. Additionally, other studies showed that ASBVd could replicate in unicellular organisms from other kingdoms, such as eukaryotic cells of the yeast *Saccharomyces cerevisae* [43] and prokaryotic cells of the cyanobacterium *Nostoc* sp. [44]. The replication of ASBVd in both unicellular organisms does not induce any phenotype, and any of these organisms represent a risk for avocado production.

## 5. Transmission

ASBVd can be transmitted to avocado via different routes. The use of diseased propagative material is the major route responsible for ASBVd spread [12]. Grafting is the principal and more effective infection technique to transmit the pathogen [11,12,45,46], as well as micrografting [47]. Natural and artificial root graft transmission between infected and healthy avocado trees have been reported [8,11], although the frequency of this transmission is unclear [12,23]. The ASBVd seed transmission was first presumed from observations of two parallel cases in California [8,48], and then in avocado seeds from symptomless carrier trees at a high rate (86–100%) of ASBVd transmission observed in symptomless seedlings. However, a low transmission rate (0–5.5%) was observed in seedlings generated from symptomatic trees [46]. The presence of the viroid was detected in the skin and pulp of avocado fruits with symptoms of sunblotch [49]. Pollen transmission of ASBVd was experimentally demonstrated using honeybees with a low transmission rate (1.8–3.1%), difficult to detect in infected orchards [49]. However, the spread of infection is more likely due to the reintroduction of infected materials than to natural processes, with an average annual growth rate of 2.3% to 4.7% of disease incidence [50]. Experiments using pruning knives indicated that ASBVd was not mechanically transmissible [8], but razor-slash inoculation was later able to successfully transmit ASBVd using sap extracted from infected trees [14]. It is important to consider that nurseries play an important role in the dissemination of ASBVd, and it is necessary to establish regulations for the production of ASBVd-free avocado plants. ASBVd uninfected avocado trees that are seed and scion donors should be the first step to obtain healthy plants free of ASBVd in order to protect the growing avocado industry.

## 6. Economic Importance

The most relevant economic impact of sunblotch disease is the effect on avocado yield. Likewise, the fruit quality and the tree growth are affected [51]. Up until today, all cultivars have been reported as susceptible to this disease. Infected trees may have a significant yield reduction when compared with healthy trees. Moreover, symptomatic fruits are of low quality, being discarded during harvesting, and the selection processes exacerbate the economic impact of this disease. Yield losses of 14% in symptomatic “Fuerte” trees and 80% in asymptomatic “Edranol” have been reported [52,53]. On the other hand, both asymptomatic “Caliente” and “Reed” trees showed yield reductions of 95% [21]. More recently, it has been reported that asymptomatic “Hass” trees have reductions of avocado yield in the range of 15–30%, while symptomatic trees are more severely affected with a reduction of 67–76%. In addition, the cost of managing this disease is very high. Removal of infected trees is expensive and, usually, the machinery is not accessible. Clearly, more information about the economic impact of this disease and its management is needed in more cultivars and in different areas. It is worth emphasizing that prevention practices are the principal way to avoid the effects of the disease, and these include the use of seeds and vegetative material free of ASBVd, the establishment of donor orchards for seed and vegetative scion production, and the continuous disinfestation of pruning, harvesting, and grafting tools [51].

## 7. Postharvest and Histological Effects of ASBVd

**Figure 3:**
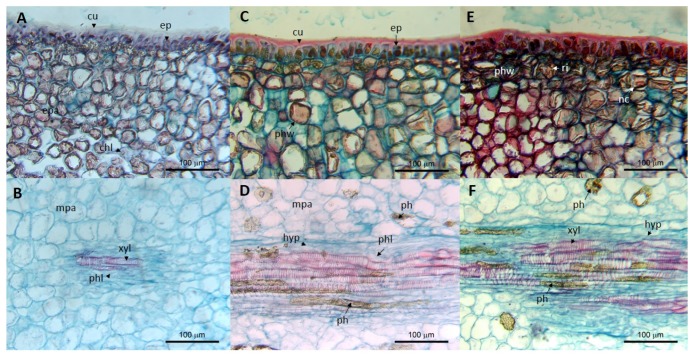
Cross-sectional micrographs of the exocarp and mesocarp tissues of avocado fruits infected with the avocado sunblotch viroid (ASBVd): (**A**,**B**) Asymptomatic; (**C**,**D**) yellowish sunken spot and (**D,E**) yellowish sunken crack. Cuticle (cu), epidermal cells (ep), exocarp parenchyma (epa), mesocarp parenchyma (mpa), chloroplasts (chl), phloem cells (phl), xylem vessels (xyl), phenol accumulation in cell wall (phw), hypertrophy (hyp), accumulation of red inclusions (ri), and necrotic cells (nc).

ASBVd symptomatic fruits are disqualified for human consumption during the packing process, generating important postharvest losses. Postharvest physiology studies about the effects of ASBVd in fruits indicate a reduction in ethylene and CO_2_ production, which causes a delay in fruit maturation, an effect that becomes more evident for symptomatic fruits [28]. Nevertheless, the weight loss, coloration changes, fruit size, mineral content and proximate analysis (protein, carbohydrates dietary fiber, and total ash) were similar in asymptomatic and control fruits. The lipid content is affected by ASBVd, but these fruits exceed the minimum required value for the quality standards (22% of dry matter and 8% oil). Symptomatic avocado fruits still fulfill the minimum requirements to be industrialized [28,54]. The ASBVd may induce anatomical and chemical changes in the cellular structure, more evident in the symptomatic tissues, including cellular disorganization, accumulation of phenolic compounds in the cytoplasm and cell walls, and reduction of chlorophyll and cytoplasmic content that may be conducive to cell collapse (Figure 3) [27,28]. Histological observations have showed organizational and chemical changes correlated to symptom severity: The parenchyma exocarp (rind) cells walls exhibited a higher accumulation of reddish polyphenols and apparent lower chloroplast content; the mesocarp parenchyma (pulp) cells reduced their cell size, and they showed disorganization and an increase in the cell number with phenolic content. Finally, the vascular tissue presented hyperplasia, phloem cells collapsed, and xylem vessels were probably occluded with phenolic compounds (Figure 3) [27,28].

## 8. Geographic Distribution

**Figure 4:**
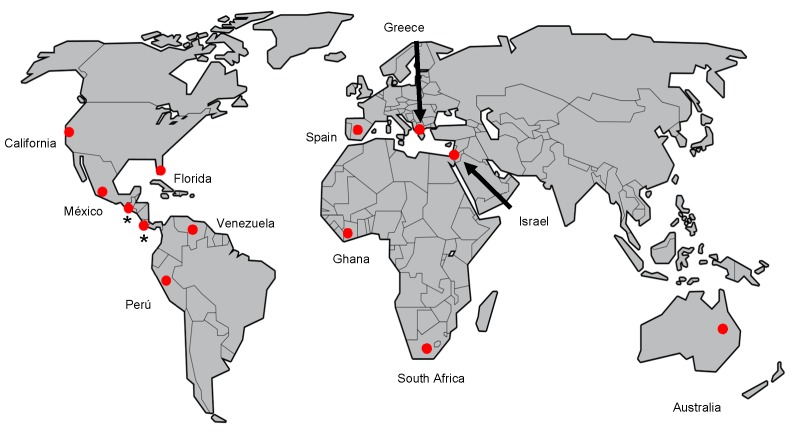
Geographical distribution of ASBVd in the five continents: America (USA, Venezuela, Perú, México), Europe (Spain, Greece), Asia (Israel), Africa (Ghana, South Africa), and Australia. 1. California 1928, 2. Florida 1939, 3. Australia 1970, 4. Venezuela 1976, 5. South Africa 1983, 6. Israel 1984, 7. Spain 1987, 8. Perú 1991, 9. Ghana 2008, 10. México 2009, 11. Greece 2018. The updated status of ASBVd in the different countries based on EPPO (2016) and Lotos et al. [65] are as follows: United States—Present, no details; Australia—Present, no details; Venezuela—Present, no details; South Africa—Present, widespread; Israel—Present, restricted distribution; Spain—Present, no details; Perú—Present, restricted distribution; Ghana—Present, few occurrences; México—Present, restricted distribution; and Greece—Present, no details. * No official reports in Guatemala or Costa Rica.

The avocado sunblotch disease occurs in areas of the five continents where avocados are grown [26,55] (Figure 4). This disease was firstly reported in the Western Hemisphere, in California, United States, as a physiological [56] or genetic [10] disorder. In 1941, Stevens and Piper reported the sunblotch disease occurred in Florida [57]. Subsequently, ASBVd was reported in the Eastern Hemisphere in New South Wales, Australia [13,58], in Venezuela [59], in the African continent (South Africa) [52], the Middle East (Israel) [60], and in Europe (Spain) [61]. Afterwards, ASBVd was confirmed in South America (Perú) [62] and in Africa (Ghana) [63], in México [64], and more recently in Greece (Crete) [65]. Although Guatemala does not have official reports of this pathogen and Costa Rica has declared its absence, The World Trade Organization (Notification of emergency measures G/SPS/N/CRI/160, 5 May 2015) and the European Plant Protection Organization [55] have reported its presence in both Guatemala and Costa Rica. Therefore, more information is needed about the official geographical distribution of ASBVd as well as more reliable and sensitive techniques to detect the pathogen.

**Figure 5:**
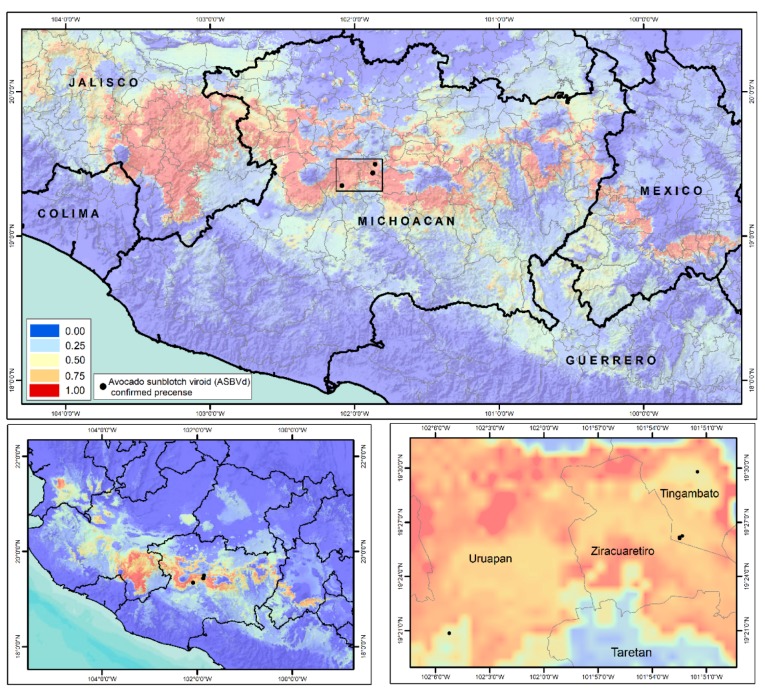
Geographical zones of greater adaptability according to maximum entropy modeling (Maxent) for establishing and developing avocado (*Persea americana* Miller) var. “Hass” in México [67] and location points (orchards) of the actual ASBVd distribution considering the reliable reports according to the ISPM 8 (International Standards for Phytosanitary Measures) [66].

The actual status of ASBVd in México is considered as “*Present, Restricted Distribution*” [55] according to the ISPM No. 8 “Determination of pest status in an area” [66]. Recent reports confirmed the presence of ASBVd in the Michoacán State in orchards located in the Tingambato Municipality (four orchards) [17,28,67] and Uruapan Municipality (one orchard) [26,54,68]. Michoacán and Jalisco are the principal states which make up the geographic region where the “Hass” avocado is cultivated (avocado belt), and the area shows high entropy values (Trans-Mexican Volcanic Belt) for the establishment of avocado, so it is important to implement major measures to prevent viroid dispersion [67] (Figure 5).

## 9. Diagnostic Methods

The sunblotch disease can be detected in avocado trees by identifying the typical symptoms in fruits; however, this approach is not applicable to infected asymptomatic trees. Therefore, diagnosis based on symptoms is not reliable and other sensitive diagnostic techniques are necessary to determine the health status of an avocado tree. Once the infectious nature of sunblotch disease was demonstrated through graft transmission [11,45], successful indexing experiments were done with avocado seedlings that showed typical symptoms in a period up to three years [8,12,23,46,69,70]. Because viroids do not encode any proteins, ELISA testing is not an option for a proper diagnosis. Instead, molecular techniques are the most reliable for a correct diagnosis. With the development of protocols to obtain preparations enriched in low-molecular-weight RNAs and the characterization of ASBVd [16,18], new and faster techniques such as polyacrylamide gel electrophoresis (PAGE) were developed [33,60]. Nevertheless, this technique was not completely reliable due to inconsistent results because known positives were often missed [71]. Improved and more reliable sensitive molecular techniques such as Reverse Transcription Polymerase Chain Reaction (RT-PCR) and dot blot hybridization have been recently adapted as better techniques to diagnose ASBVd-infected avocado plants.

Molecular techniques such as dot blot hybridization with radioactively labelled complementary cDNA [72] and digoxigenin-labelled complementary RNA [37] have been developed as alternative methods to detect ASBVd. Likewise, digoxigenin- and biotin-labelled probes were also used, coupled with electron microscopy, for detecting ASBVd in the chloroplasts [73,74,75]. In situ hybridization using RNA complementary probes both digoxigenin- and biotin-labelled [74] is an additional tool to detect ASBVd in the chloroplast. Although these techniques of dot blot hybridization are more sensitive than PAGE, they are not completely reliable because known positives were often not detected.

RT-PCR performed in one [18] or two steps [24,76] has long been used as a routine laboratory test for ASBVd. More recently, a more sensitive method based on real-time RT-PCR was developed [71]. Non-destructive massive detection of avocado trees showing symptoms of sunblotch disease was performed using satellite spectral imaging [68] with an accuracy of 70% of actual ASBVd infection with respect to parallel analyses performed using RT-PCR [68]. Although this technique provides an additional tool to detect ASBVd-infected plants, more research in different regions is needed to corroborate these results.

## 10. Management of ASBVd

Avocado cultivars might have a different response against the ASBVd infection, but there are no therapeutic or curative methods to control sunblotch disease [51]. Therefore, exclusion is the most effective way to manage the disease, and the availability of ASBVd-free plant material from certified nurseries is the most important requirement to avoid spreading the pathogen. Asymptomatic infected trees play an important role in the epidemiology of the pathogen because they are the principal source for viroid dispersion through budding or grafting practices [51]. Additionally, neighboring trees (15 m radius) also need to be destroyed to impede root-grafting [30]. The agronomic management of avocado is the principal route for disease dispersion in commercial orchards. In contrast, the native populations of Mexican avocado (*P. americana* var. *drymifolia*) remain healthy due to the scarce manipulation or vegetative propagation [67]. Despite ASBVd being transmitted by seed, asymptomatic fruits can be exported since they are for human consumption rather than for propagation. Thus, the material used to establish new orchards needs to be obtained from certified ASBVd-free sources. Reliable methods to diagnose ASBVd from trees providing seeds and scions must be used in all nurseries [51]. Disinfestation of pruning tools and harvesting and grafting material with sodium hypochlorite (1.5%) is a simple but crucial step to maintain uninfected avocado trees and avoid the spread of an invisible foe of avocado.

## 11. Future perspectives

This review emphasizes the importance of ASBVd in the avocado industry. Strategies for ASBVd management need to be implemented. The use of ASBVd-free avocado plants from certified nurseries needs to be encouraged, and regulatory actions must regulate the movement of uncertified avocado material to avoid ASBVd spread.
